# Myricitrin Protects against Doxorubicin-Induced Cardiotoxicity by Counteracting Oxidative Stress and Inhibiting Mitochondrial Apoptosis via ERK/P53 Pathway

**DOI:** 10.1155/2016/6093783

**Published:** 2016-09-14

**Authors:** Jing Sun, Guibo Sun, Xiaolan Cui, Xiangbao Meng, Meng Qin, Xiaobo Sun

**Affiliations:** ^1^Institute of Chinese Materia Medica, China Academy of Chinese Medical Sciences, Chaoyang District, Beijing 100029, China; ^2^Key Laboratory of Bioactive Substances and Resources Utilization of Chinese Herbal Medicine, Ministry of Education, Institute of Medicinal Plant Development, Chinese Academy of Medical Sciences and Peking Union Medical College, Haidian District, Beijing 100193, China

## Abstract

Doxorubicin (Dox) is one of the most effective and widely used anthracycline antineoplastic antibiotics. Unfortunately, the use of Dox is limited by its cumulative and dose-dependent cardiac toxicity. Myricitrin, a natural flavonoid which is isolated from the ground bark of* Myrica rubra*, has recently been found to have a strong antioxidative effect. This study aimed to evaluate the possible protective effect of myricitrin against Dox-induced cardiotoxicity and the underlying mechanisms. An in vivo investigation in SD rats demonstrated that myricitrin significantly reduced the Dox-induced myocardial damage, as indicated by the decreases in the cardiac index, amelioration of heart pathological injuries, and decreases in the serum cardiac enzyme levels. In addition, in vitro studies showed that myricitrin effectively reduced the Dox-induced cell toxicity. Further study showed that myricitrin exerted its function by counteracting oxidative stress and increasing the activities of antioxidant enzymes. Moreover, myricitrin suppressed the myocardial apoptosis induced by Dox, as indicated by decreases in the activation of caspase-3 and the numbers of TUNEL-positive cells, maintenance of the mitochondrial membrane potential, and increase in the Bcl-2/Bax ratio. Further mechanism study revealed that myricitrin-induced suppression of myocardial apoptosis relied on the ERK/p53-mediated mitochondrial apoptosis pathway.

## 1. Introduction

Doxorubicin (Dox), an anthracycline chemotherapeutic agent, is widely used in treatment of a broad range of solid tumors and malignant hematologic diseases. However, its clinical use is often limited by its dose-related cardiotoxicity, which may eventually progress to heart failure [1, 2]. Accordingly, identification of a safe and effective antagonist to this serious side effect of Dox remains a key research issue in both cardiology and oncology. The exact mechanism of the Dox-induced cardiotoxicity and its progression to heart failure still remains unknown. However, additional evidence has shown that increased oxidative stress and cardiomyocyte apoptosis play important roles in the Dox-induced cardiotoxicity [3, 4]. Due to its unique chemical structure, Dox is likely to generate reactive oxygen species (ROS) by redox cycling. In addition, large doses and/or long-term use of Dox decreases the levels of the endogenous antioxidant enzymes that are responsible for scavenging the ROS [5, 6]. Under these conditions, ROS are generated, and oxidative stress, which is characterized by an imbalance between prooxidants and antioxidants, is stimulated. Oxidative stress directly or indirectly activates several signaling pathways that leads to cardiomyocyte apoptosis and, ultimately, to heart failure [7, 8]. Therefore, antioxidative and antiapoptotic therapeutic agents are the two major means of addressing the cardiotoxicity induced by Dox.

Apoptosis is stimulated by two typical signaling pathways, specifically, the mitochondrial pathway and the death receptor pathway [9]. Our previous studies indicated that the mitochondrial pathway, rather than the death receptor pathway, is the main mechanism that participates in Dox-induced cardiomyocyte apoptosis [10]. The initiation of mitochondrial apoptosis in Dox-induced cardiotoxicity has been reported to involve the ERK/p53 signal transduction pathway [11]. Extracellular signal-regulated kinase (ERK) is phosphorylated after Dox treatment, and this is followed by the activation of tumor suppressor gene p53, which then translocates to the nucleus [12]. The increased expression of p53 is associated with Bcl-2 family genes, which trigger the collapse of the mitochondrial membrane potential, cytochrome c release, activation of caspase-9 and caspase-3, and, ultimately, cell death via apoptosis [13, 14].

**Figure 1:**
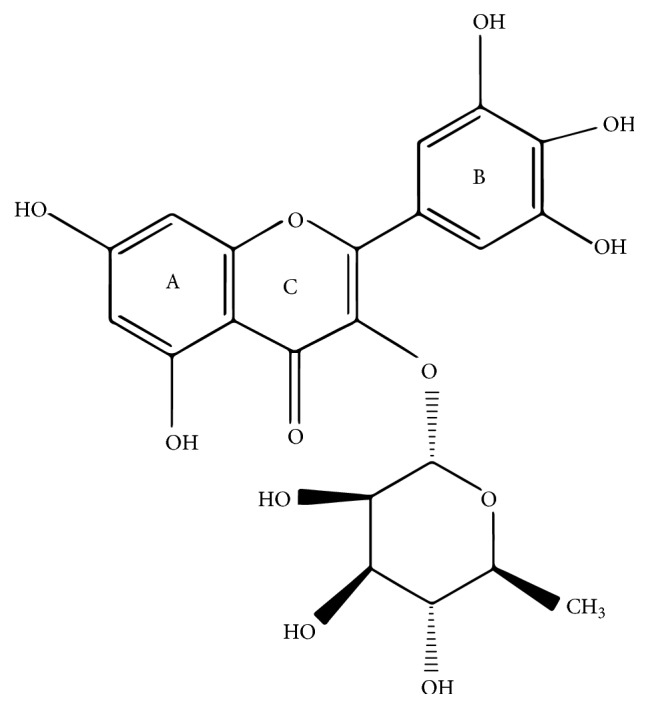
Molecular structure of myricitrin.

In recent years, considerable research efforts have been focused on flavonoids, a group of naturally occurring compounds that exhibit a wide range of biological activities. Many studies have shown that flavonoids are major components of traditional Chinese herbal which are frequently used in the prevention and treatment of cardiovascular diseases. Myricitrin (Figure 1) is a natural flavonoglycoside that is extracted from the fruits, leaves, branches, and bark of* Myrica rubra* or other natural plants, such as* Ampelopsis grossedentata*,* Chrysobalanus icaco*, and* Nymphaea lotus* [15–17]. Myricitrin reportedly shows various beneficial activities, including antiallergic, anti-inflammatory, antinociception, and anxiolytic effects as well as other biological activities [18–20]. Moreover, data from our previous study demonstrated that myricitrin inhibited oxidative stress-induced endothelial damage and atherosclerosis [21, 22]. Nevertheless, little is known about the direct protective effect of myricitrin against Dox-induced cardiotoxicity or about the underlying mechanisms.

The present study investigated the protective role of myricitrin in Dox-induced cardiotoxicity in vivo and in vitro. In a rat model of chronic Dox-induced cardiotoxicity, myricitrin (2.5 and 5 mg/kg, i.p.) treatment for 7 days attenuated the Dox-induced myocardial damage via a decrease in ROS production and a reduction in the level of apoptosis of myocardial cells. Moreover, we evaluated the cardioprotective mechanism of myricitrin at the molecular level. Myricitrin treatment can inhibit the activation of the mitochondrial apoptosis pathway, including stabilizing the mitochondrial transmembrane potential (Δ*ψ*_*m*_), reducing the release of cytochrome c from the mitochondria, and inhibiting the activation of caspase-9 and caspase-3. It also significantly modulated the expression of Bcl-2 family proteins by inhibiting the ERK/p53 pathways, which resulted in a decrease in the apoptosis of the cardiomyocytes. These findings suggested that myricitrin may have potential as a candidate drug for the treatment of Dox-induced cardiotoxicity.

## 2. Materials and Methods

### 2.1. Reagents

Dulbecco's modified Eagle's medium (DMEM) and fetal bovine serum were purchased from Gibco Life Technologies (NY, USA). Doxorubicin was purchased from Shenzhen Main Luck Pharmaceuticals, Inc. (Shenzhen, China). The Cell Counting Kit-8 was obtained from Dojindo Laboratory (Japan). The fluorescent dye JC-1 was purchased from Sigma-Aldrich (St. Louis, MO, USA). The kits for measuring lactate dehydrogenase (LDH), creatine kinase (CK), and aspartate aminotransferase (AST) were obtained from Biosino Bio-Technology and Science Incorporation (Hong Kong, China). The kits for determining the levels of malondialdehyde (MDA) and the superoxide dismutase (SOD), catalase (CAT), and glutathione peroxidase (GSH-Px) activities were purchased from the Nanjing Jiancheng Institute of Biological Engineering (Nanjing, China). The caspase-3 activity fluorometric assay kit was purchased from BioVision (Palo Alto, CA, USA). The terminal deoxynucleotidyl transferase-mediated dUTP nick end-labeling (TUNEL) assay kit was purchased from Roche Diagnostics GmbH (Mannheim, Germany). Primary antibodies against *β*-actin, cleaved caspase-3, cleaved caspase-9, cyt-c, Bcl-2, Bcl-xl, Bax, Bad, p53, total ERK1/2, p-ERK1/2, total JNK, and p-JNK were obtained from Santa Cruz Biotechnology (Santa Cruz, CA, USA). The horseradish peroxidase-conjugated goat anti-mouse or goat anti-rabbit IgG secondary antibodies were purchased from CW Biotech (Beijing, China). The purity of all other chemicals was at least of analytical grade.

### 2.2. Preparation of Myricitrin

Myricitrin was isolated from the air-dried and ground barks of* Myrica rubra*, which was identified by Professor Rui-le Pan (Institute of Medicinal Plant Development, Beijing, China). The specific preparation process was consistent with the description by Professor Sun et al. [21]. The purity of the product was over 99% as measured by high-performance liquid chromatography.

### 2.3. Animals

Male Sprague-Dawley rats (weighing 290–310 g, 8 weeks of age) were purchased from Vital River Laboratories, Beijing, China. The animals were kept under controlled laboratory conditions (temperature: 22°C ± 1°C, relative humidity: 60%, and a 12 h light/dark cycle) and allowed free access to standard food and water. All interventions and animal care procedures were approved by the Animal Ethics Committee of Peking Union Medical College and conformed to the Guide for the Care and Use of Laboratory Animals published by the US National Institutes of Health (NIH), Publication number 8023, revised 1978.

### 2.4. Experimental Protocols

All rats were assigned to one of six groups: (1) control group (Cont, *n* = 16): the rats were intraperitoneally (i.p.) injected with saline; (2) Dox group (Dox, *n* = 16): the rats were dosed (i.p.) with Dox (3 mg/kg every other day for a total of 3 times) [10]; (3) myricitrin high-dose group (Myr5, *n* = 16): the rats were treated (i.p.) with myricitrin (5 mg/kg every day) for 7 days; (4) myricitrin low-dose group (Myr2.5, *n* = 16): the rats were treated (i.p.) with myricitrin (2.5 mg/kg every day) for 7 days; (5) myricitrin high-dose combined with Dox group (Dox + Myr5, *n* = 16): the rats were pretreated with myricitrin at a dose of 5 mg/kg i.p. for 7 days prior to Dox; and (6) myricitrin low-dose combined with Dox group (Dox + Myr2.5, *n* = 16): the rats were pretreated with myricitrin at a dose of 2.5 mg/kg i.p. for 7 days prior to Dox. The rats were sacrificed after 28 days at which time the hearts and blood were collected for histopathological examination, biochemical measurements, and other analyses.

### 2.5. Heart Histopathological Examination

The left ventricles of the hearts were fixed in 4% paraformaldehyde, then trimmed, and embedded in paraffin blocks. Paraffin sections (3 mm) were cut, stained with hematoxylin and eosin (HE), and examined by light microscopy (CKX41, Olympus, Tokyo, Japan) to evaluate the histopathological changes of the hearts.

### 2.6. Measurements of Biochemical Variables

The activities of LDH, CK, and AST in the serum, which were used as key diagnostic indicators of myocardial injury [23], were measured using kits (Nanjing Jiancheng Biotechnology Institute, China). The MDA content and superoxide dismutase (SOD), catalase (CAT), and glutathione peroxidase (GSH-Px) activities were measured to evaluate the antioxidant activities of myricitrin in hearts according to the kit manufacturer's instructions [6] (Nanjing Jiancheng Biotechnology Institute, China).

### 2.7. Transmission Electron Microscopy

The left ventricles of the hearts were fixed in 4% glutaraldehyde in 0.1 M cacodylate buffer and postfixed with 1% osmium tetroxide in the same buffer, followed by dehydration, and embedded in Epon. Sections (50 nm) were cut and collected for transmission electron microscopy at an acceleration voltage of 60 kV as previously described [24].

### 2.8. Cell Culture

Rat embryonic cardiomyoblast-derived H9c2 cardiomyocytes were obtained from the Cell Bank of the Chinese Academy of Sciences (Shanghai, China). The H9c2 cardiomyocytes were grown in Dulbecco's modified Eagle's medium (DMEM) supplemented with 10% (v/v) fetal bovine serum, 100 U/mL penicillin (Sigma, St. Louis, MO), 100 *μ*g/mL streptomycin (Sigma, St. Louis, MO), and 2 mM L-glutamine. The cells were maintained at 37°C in a humidified incubator containing 5% CO_2_. The medium was changed every day. The H9c2 cardiomyocytes were plated at an appropriate density according to the experimental design and were grown to 80% confluence before the drug exposures in the various experiments.

### 2.9. Analysis of Cell Viability and LDH Release

The H9c2 cardiomyocytes were treated with the vehicle (0.1% DMSO) or myricitrin (3.125, 6.25, 12.5, and 25 *μ*g/mL) for 12 h in presence or absence of exposure to Dox (1 mM) for an additional 36 h. The cell viability was evaluated using the Cell Counting Kit-8 (CCK8) assay according to the manufacturer's manual. The medium was collected for the measurement of the lactate LDH release using a LDH assay kit.

### 2.10. Assessment of ROS Production, MDA Levels, and Activities of SOD, CAT, and GSH-Px in H9c2 Cardiomyocytes

The intracellular ROS production was monitored using an automatic microplate reader (Spectrafluor, TECAN, Sunrise, Austria) with an emission at 525 nm and an excitation of 495 nm. The cells were harvested, ultrasonicated, and centrifuged at 1000 rpm at 4°C for 5 min; then the levels of MDA and activities of SOD, CAT, and GSH-Px in the supernatant were assessed with the corresponding detection kits.

### 2.11. In Situ Detection of Apoptosis

The DNA fragmentation in the heart tissues and H9c2 cardiomyocytes was evaluated using the TUNEL assay with the In Situ Apoptosis Detection Kit. The deparaffinized and rehydrated heart slices or fixed H9c2 cardiomyocytes were incubated with proteinase K (20 mg/mL) at room temperature for 15 min, then rinsed with the equilibration buffer, and incubated with the working-strength terminal deoxynucleotidyl transferase enzyme at 37°C for 1 h in a humidified chamber. After rinsing with a stop/wash buffer, the sections were incubated with the working-strength anti-digoxigenin conjugate for another 30 min. The slices were stained with 4′6-diamidino-2-phenylindole and visualized by fluorescence microscope (DM4000B, Leica Wetzlar, Germany). The apoptotic cells were counted in at least 100 cells from three randomly selected fields in each treatment and expressed as a percentage of the total number of cells counted [25].

### 2.12. Analysis of Caspase-3 and Caspase-9 Activation

The Caspase-3 and Caspase-9 activities were measured by a fluorometric assay kit (BioVision, Mountain View, CA, USA) according to the manufacturer's instructions. The samples were read in a microplate reader (Spectrafluor, TECAN, Sunrise, Austria) using an excitation wavelength of 400 nm and an emission wavelength of 505 nm.

### 2.13. Determination of the Mitochondrial Membrane Potential (Δ*ψ*_*m*_)

5,5′,6,6′-Tetrachloro-1,1′,3,3′-tetraethylbenzimidazolyl-carbocya-nine iodide (JC-1, Invitrogen) was used to monitor the changes in the mitochondrial membrane potential (Δ*ψ*_*m*_). The harvested H9c2 cardiomyocytes were loaded with 2 *μ*M JC-1 in the dark at 37°C for 30 min, washed twice with PBS, and viewed using a fluorescence microscope (Leica, Germany).

### 2.14. Protein Extraction and Western Blot Analysis

The cultured H9c2 cardiomyocytes were harvested and washed once with PBS, and then the cytoplasmic fractions were isolated using lysis buffer containing 1% protease inhibitors on ice. The lysates were centrifuged at 12,000 ×g at 4°C for 15 min, and the protein concentrations in the resulting supernatant were determined using the bicinchoninic acid assay. Equal amounts (15 *μ*g) of the protein fractions were separated using 10% sodium dodecyl sulfate polyacrylamide gels (SDS-PAGE) and transferred onto nitrocellulose membrane in Tris-glycine buffer at 110 V for 1 h. The membranes were blocked with 5% (w/v) nonfat milk powder for 1 h in Tris-buffered saline containing 0.1% (v/v) Tween-20 (TBST). Then, the membranes were incubated with the various primary antibodies at 4°C overnight. After washing three times with TBST and incubating with the secondary antibodies for 2 h, the signals on the membranes were visualized using enhanced chemiluminescence.

### 2.15. Statistical Analyses

The results are expressed as the means ± standard deviations. All statistical analyses were performed with Student's *t*-test or ANOVA using Prism 5.00 software. *P* values less than 0.05 were considered significant.

## 3. Results

### 3.1. Myricitrin Prevented the Dox-Induced Cardiac Injury In Vivo

**Figure 2:**
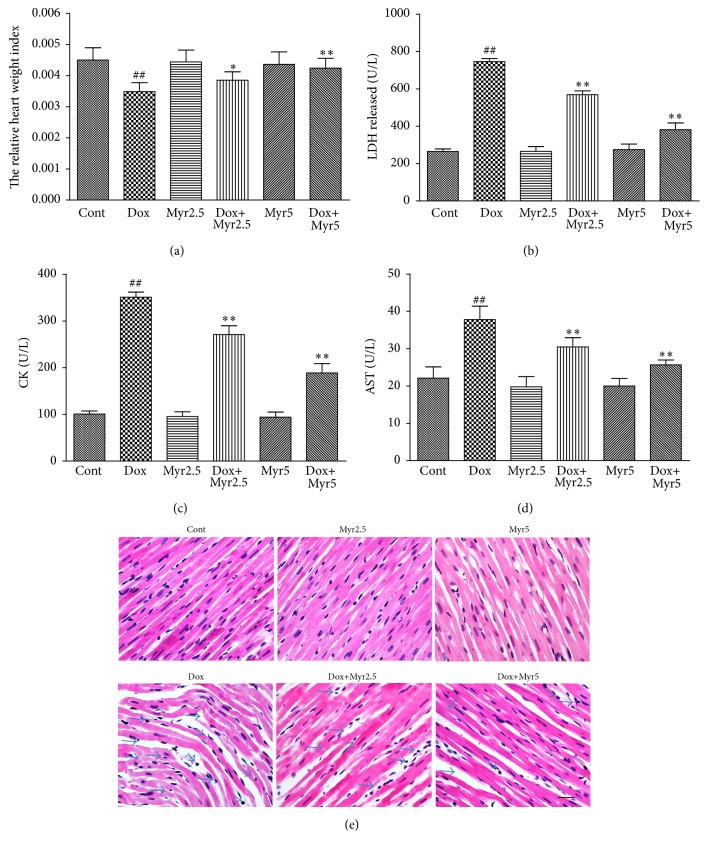
Effects of myricitrin on the Dox-induced cardiotoxicity in vivo. The rats were intraperitoneally (i.p.) treated with Dox (3 mg/kg every other day for a cumulative dose of 9 mg/kg) in presence or absence of myricitrin (2.5 and 5 mg/kg i.p. before Dox administration). After 28 days, the relative heart weight index (heart weight/body weight ratio g/g) was measured (a). The effects of myricitrin on the LDH (b), CK (c), and AST (d) levels were measured according to the kit manufacturer's instructions. The effects of myricitrin on the histological changes in the heart tissues were evaluated using HE staining. Solid arrow indicated the abnormal phenotypes including disorganization of myofibrillar arrays, cytoplasmic vacuolization, and intense infiltration with neutrophil granulocytes. Scale bar: 500 *µ*m (e). The values (*n* = 8 per group) are expressed as the means ± SD. ^##^ *P* < 0.01 versus Cont; ^*∗*^ *P* < 0.05 and ^*∗∗*^ *P* < 0.01 versus Dox.

The cardiotoxicity of Dox and the protective effect of myricitrin in rats were evaluated first. The rats were intraperitoneally injected with Dox (3 mg/kg every other day for a cumulative dose of 9 mg/kg). As shown in Figure 2(a), after 28 days, the ratio of the heart weights to body weights decreased significantly. In contrast, pretreatment of the rats with myricitrin (2.5 mg/kg and 5 mg/kg before Dox administration) prevented these changes in the heart weight/body weight ratio. Pretreatment with myricitrin also clearly attenuated the Dox-induced increases in the serum cardiac enzymes levels in a dose-dependent manner. None of the rats treated with myricitrin alone at either concentration showed any obvious abnormalities compared with the controls (Figures 2(b), 2(c), and 2(d)). The morphological changes associated with myocardial damage were also evaluated using HE staining. No obvious abnormalities were observed in control and myricitrin-alone groups, whereas serious myocardial damage characterized by disorganization of myofibrillar arrays, cytoplasmic vacuolization, and intense infiltration of neutrophil granulocytes was observed in the Dox group, as shown in Figure 2(e). Myricitrin pretreatment appeared to prevent the pathological injuries.

### 3.2. Myricitrin Protected H9c2 Rat Cardiomyocytes from Cytotoxicity

**Figure 3:**
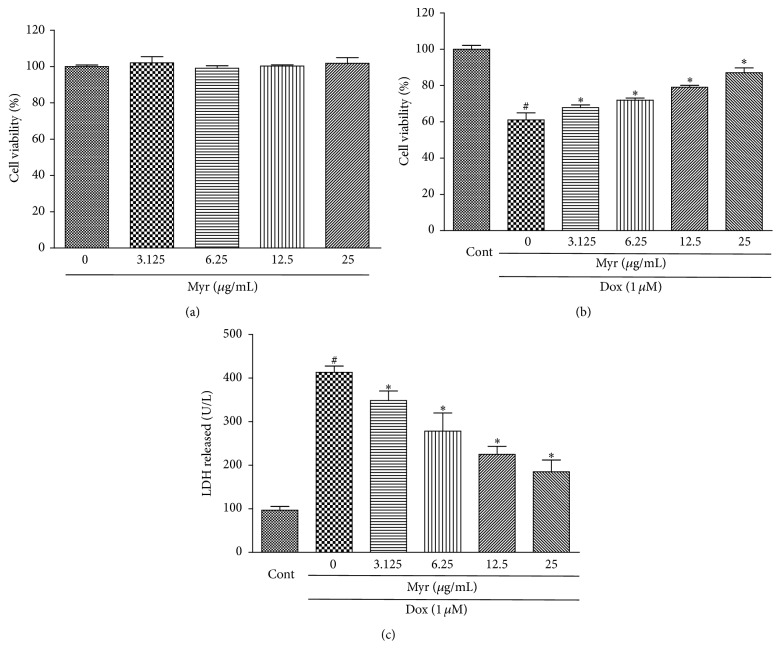
Effects of myricitrin on Dox-induced cardiotoxicity in vitro. The H9c2 cells were treated with various concentrations of myricitrin (3.125, 6.25, 12.5, and 25 *μ*g/mL) for 12 h, and the cell viability was determined using the CCK8 assay and expressed as the percentage relative to the control group (a). The control and myricitrin-treated cells were subsequently exposed to 1 *μ*M Dox for an additional 36 h, and the cell viability (b) and LDH release were measured (c). All data are expressed as the means ± SD (*n* = 3). ^#^ *P* < 0.05 versus Cont; ^*∗*^ *P* < 0.05 versus Dox.

The cytotoxicity of Dox and the protective effect of myricitrin on the H9c2 cardiomyocytes were evaluated using the CCK8 and LDH assays. As shown in Figure 3(a), H9c2 rat cardiomyocytes were pretreated with different myricitrin concentrations (3.125, 6.25, 12.5, and 25 *μ*g/mL) for 12 h, followed by 36 h of 1 *μ*M Dox treatment [26]. Pretreatment of myricitrin at various concentrations protected H9c2 cells from Dox-induced cytotoxicity in a dose-dependent manner. Myricitrin significantly increased the cell viability from 61.1% ± 3.8% up to 87.1% ± 2.6% at 25 *μ*g/mL. Treatment with myricitrin at different concentrations only had no toxicity on cell viability (Figure 3(b)). These results suggested that myricitrin protected H9c2 rat cardiomyocytes from Dox-induced cell injuries. Myricitrin at 25 *μ*g/mL showed the highest protective effect. Preincubation with myricitrin (3.125, 6.25, 12.5, and 25 *μ*g/mL) also prevented LDH leakage induced by Dox in a dose-dependent manner (Figure 3(c)).

### 3.3. Myricitrin Ameliorated the Dox-Induced Apoptotic Damage In Vivo and In Vitro

**Figure 4:**
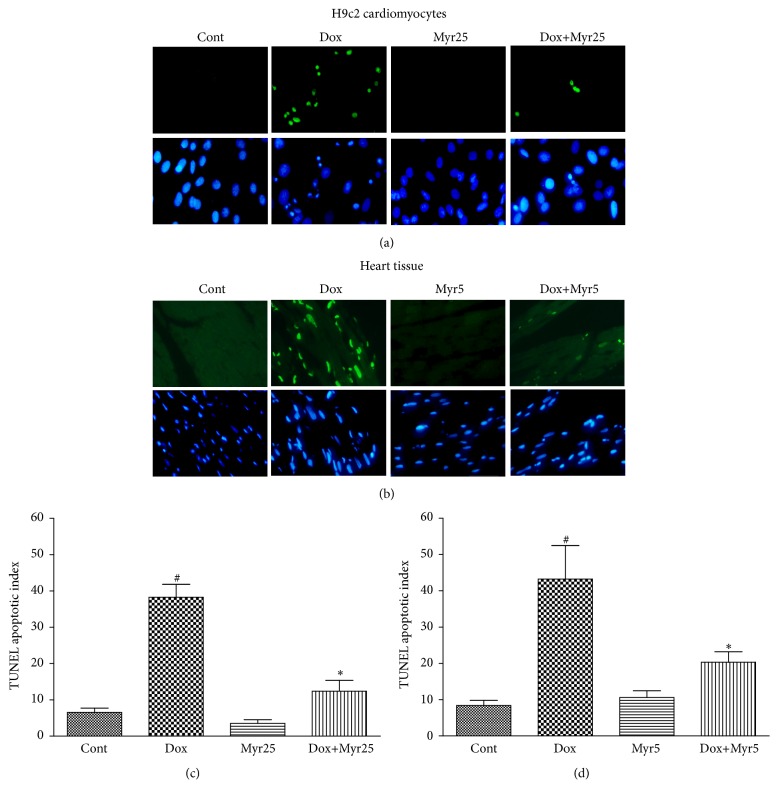
Effects of Dox and myricitrin on apoptosis in vivo and in vitro. Internucleosomal DNA fragmentation of H9c2 cardiomyocytes and heart tissues was determined using the TUNEL assay ((a) and (b)). The TUNEL apoptotic index was determined by calculating the ratio of TUNEL-positive cells to total cells ((c) and (d)). All data are expressed as the means ± SD (*n* = 3 per group). ^#^ *P* < 0.05 versus Cont; ^*∗*^ *P* < 0.05 versus Dox.

Apoptotic damage is important in the pathogenesis of cardiovascular diseases and contributes to the development of the cardiotoxicity induced by Dox. We therefore first evaluated whether the protective effect of myricitrin was associated with apoptosis. As shown in Figures 4(a) and 4(b), the TUNEL assay revealed that myricitrin pretreatment prevented the severe DNA fragmentation induced by Dox in the H9c2 cardiomyocytes and rat heart tissues. The percentage of TUNEL-positive cells was also calculated and is presented in the histograms (Figures 4(c) and 4(d)). These results indicated that mechanism for the myricitrin-mediated protection from the cardiotoxicity caused by Dox involved apoptosis to a certain degree.

### 3.4. Myricitrin Prevented the Dox-Induced Oxidative Stress In Vivo and In Vitro

**Figure 5:**
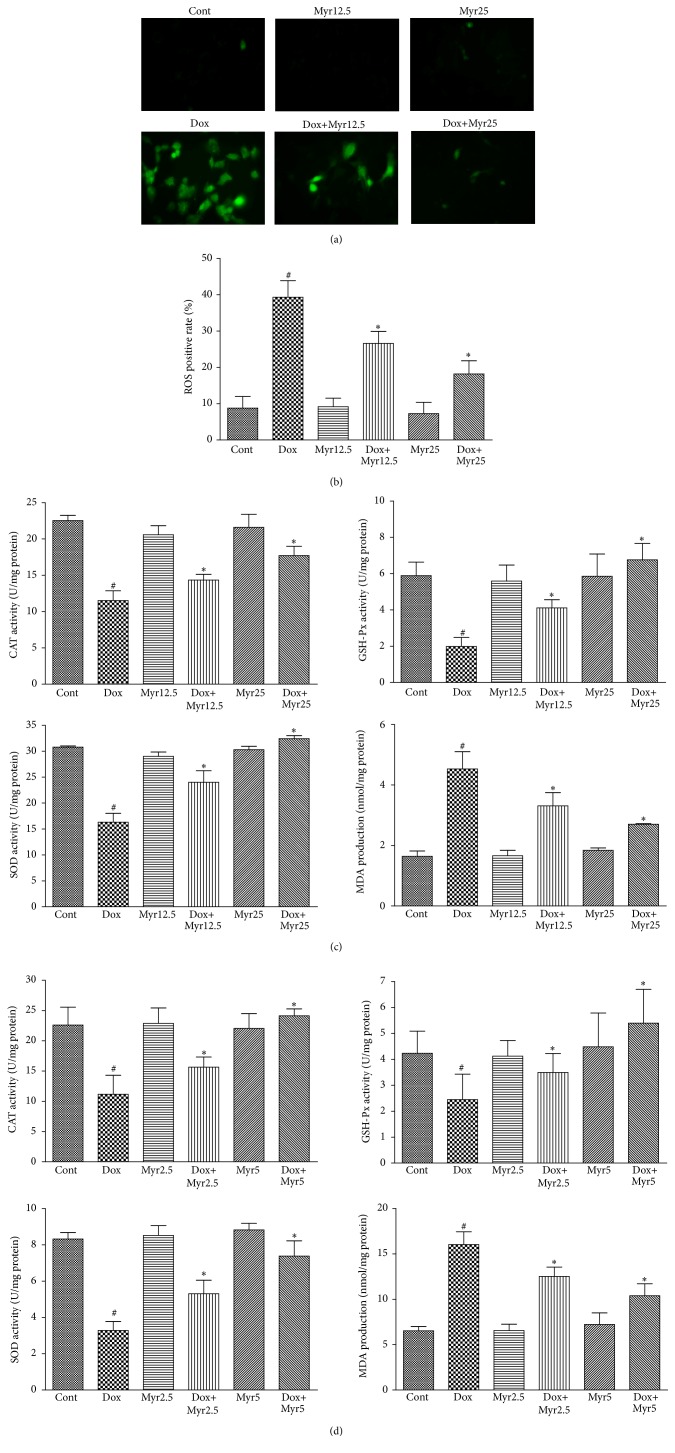
Effects of Dox and myricitrin on the antioxidant capacity in vivo and in vitro. The H9c2 cardiomyocytes were treated with Dox for 36 h in presence or absence of myricitrin (12.5 and 25 *μ*g/mL). The ROS production was visualized using fluorescence microscopy and quantified using an automatic microplate reader ((a) and (b)). The effects of myricitrin on the activities of CAT, GSH-Px, and SOD and the levels of MDA in the H9c2 cells (c) and rat heart tissues (d) were measured using the corresponding detection kits. All data are expressed as the means ± SD (*n* = 3–8). ^#^ *P* < 0.05 versus Cont; ^*∗*^ *P* < 0.05 versus Dox.

A previous study demonstrated that Dox increases the production of ROS, which leads to cell apoptosis [27]. Therefore, we investigated the ROS generation in response to Dox stimulation and its regulation by myricitrin using a total ROS detection kit. As shown in Figures 5(a) and 5(b), a remarkable increase in the intracellular ROS production compared with the control was detected after 36 h of Dox treatment, whereas pretreatment with myricitrin significantly decreased the generation of ROS. Activities of antioxidant enzyme including CAT, SOD, GSH-Px, and lipid peroxide as MDA were also taken for the indicators of oxidative stress. Thus, we measured the effects of Dox and myricitrin on these indicators in vivo and in vitro. As shown in Figures 5(c) and 5(d), Dox caused decreases in the SOD, CAT, and GSH-Px activities and an increase in the MDA production in both H9c2 cardiomyocytes and rat serum, whereas all of these changes were effectively ameliorated by myricitrin in a dose-dependent manner.

### 3.5. Myricitrin Reversed the Dox-Induced Mitochondrial Dysfunction and Cytochrome c Release In Vivo and In Vitro

**Figure 6:**
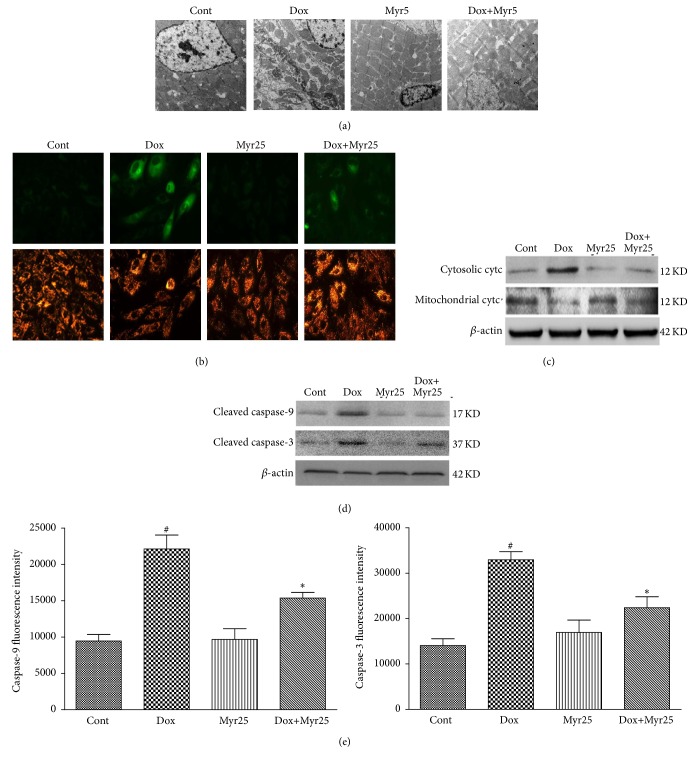
Effects of Dox and myricitrin on the mitochondrial apoptotic pathway in vivo and in vitro. Mitochondrial injury in the rat heart tissues was evaluated using transmission electron microscopy (a). H9c2 cells stained with JC-1 dye were visualized by fluorescence microscopy (b). Expressions of cytosolic cytc and mitochondrial cytc in H9c2 cardiomyocytes were determined using western blotting analysis (c). Production of cleaved caspase-9 and caspase-3 was determined using western blotting analysis (d). Caspase-3 and caspase-9 activities were measured using a fluorometric assay kit in the H9c2 cardiomyocytes (e). All data are expressed as the means ± SD (*n* = 3–8 per group). ^#^ *P* < 0.05 versus Cont; ^*∗*^ *P* < 0.05 versus Dox.

Mitochondrial dysfunction, including mitochondrial potential (Δ*ψ*_*m*_) depolarization and subsequent cytochrome c release, plays crucial roles in the Dox-induced apoptotic damage [28]. Mitochondrial function in vivo was therefore evaluated using transmission electron microscopy. As shown in Figure 6(a), Dox treatment caused myofibrillar fracture and loss, mitochondrial swelling, cytoplasmic vacuolization, chromatin condensation, and cardiomyocyte necrosis in the rat heart tissues. Myricitrin mitigated this structural damage as expected. Furthermore, JC-1 was used to assess the Δ*ψ*_*m*_ in H9c2 cardiomyocytes. The monomeric form of JC-1 in the cytosol emits a green fluorescence, and aggregates of the dye in the mitochondria of normal cells emit a red fluorescence. However, in apoptotic cells, JC-1 emits only the green fluorescence from the cytosol. As shown in Figure 6(b), the Dox-induced Δ*ψ*_*m*_ depolarization was mitigated by myricitrin pretreatment. In addition, myricitrin prevented the Dox-induced cytochrome c release from the mitochondria into the cytosol as indicated by an immunoblotting assay (Figure 6(c)). In addition, caspase-3 and caspase-9, two major biomarker of mitochondrial apoptosis [29], were also tested by immunoblotting assay and fluorescein active staining kits. As shown in Figures 6(d) and 6(e), myricitrin inhibited the activation of caspase-3/caspase-9 in H9c2 cardiomyocytes and decreased the production of cleaved caspase-3/caspase-9.

### 3.6. Involvement of ERK1/2 and JNK Phosphorylation in Myricitrin-Mediated Inhibition of Caspase-3 Activation and Cytotoxicity

**Figure 7:**
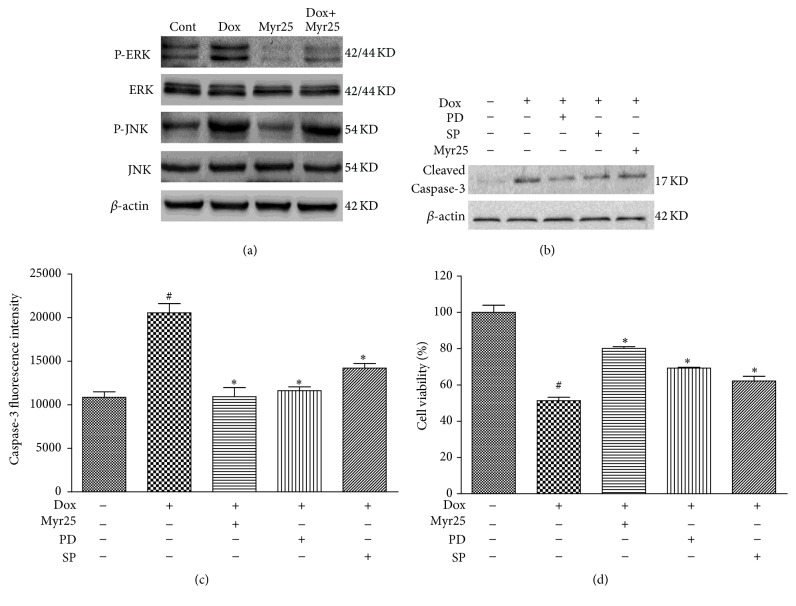
Involvement of ERK1/2 and JNK phosphorylation in myricitrin-mediated inhibition of caspase-3 activation and cytotoxicity. The H9c2 cardiomyocytes were pretreated with or without myricitrin (25 *μ*g/mL) for 12 h prior to Dox exposure. The phosphorylation of ERK1/2 and JNK was detected using immunoblotting (a). The effects of SP600125 (a JNK-specific inhibitor) and PD98059 (an ERK-specific inhibitor) on the myricitrin-mediated inhibition of cleaved caspase-3 production and cytotoxicity in H9c2 cardiomyocytes were measured ((b), (c), and (d)). All data are expressed as the means ± SD (*n* = 3). ^#^ *P* < 0.05 versus Cont; ^*∗*^ *P* < 0.05 versus Dox.

The ERK1/2 and JNK signaling pathways have been reported to play key roles in protecting cells from the apoptosis caused by Dox [11, 30]. Therefore, we first evaluated the expression of both total and phosphorylated ERK1/2 and JNK proteins using western blotting analysis. As shown in Figure 7(a), Dox treatment promoted expression of P-ERK1/2 and P-JNK whereas pretreatment with myricitrin significantly inhibited the phosphorylation of ERK1/2 and JNK. Furthermore, pretreatment with PD98059 (an ERK-specific inhibitor) and SP600125 (a JNK-specific inhibitor) attenuated the decline of cell viability and the production of cleaved caspase-3 that were induced by Dox (Figures 7(b), 7(c), and 7(d)). These results suggested that the ERK1/2 and JNK signaling pathways were involved in the antiapoptotic and cytoprotective effects of myricitrin.

### 3.7. Myricitrin Modulated the Expression of the Bcl-2 Family Proteins (Bcl-2, Bcl-xl, Bad, and Bax) and p53 in H9c2 Rat Cardiomyocytes

**Figure 8:**
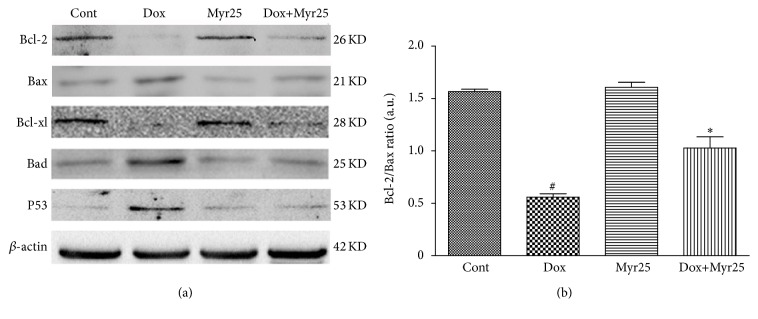
Effects of Dox and myricitrin on expression of Bcl-2 family proteins and P53 in H9c2 cardiomyocytes. The expression levels of Bcl-2, Bax, Bcl-xl, and Bad were determined using an immunoblotting assay (a). The Bcl-2/Bax ratio was calculated (b). All data are expressed as the means ± SD (*n* = 3). ^#^ *P* < 0.05 versus Cont; ^*∗*^ *P* < 0.05 versus Dox-treated cells.

The Bcl-2 family of proteins, which includes Bcl-2, Bcl-xl, Bad, and Bax, is involved in the regulation of apoptosis. The effects of myricitrin on the expression levels of Bcl-2 protein family members in H9c2 rat cardiomyocytes were evaluated using western blotting analysis. As shown in Figure 8(a), Dox caused a significant upregulation of Bax and Bad and a downregulation of Bcl-2 and Bcl-xl. Myricitrin pretreatment prevented these effects. Similarly, preincubation with myricitrin reduced the Dox-induced increase in p53 expression. In addition, myricitrin prevented the Dox-induced change in the calculated Bcl-2/Bax ratio (Figure 8(b)).

### 3.8. Myricitrin Modulated the Bcl-2 and Bax Expression through the ERK1/2-p53 Pathway in H9c2 Rat Cardiomyocytes

**Figure 9:**
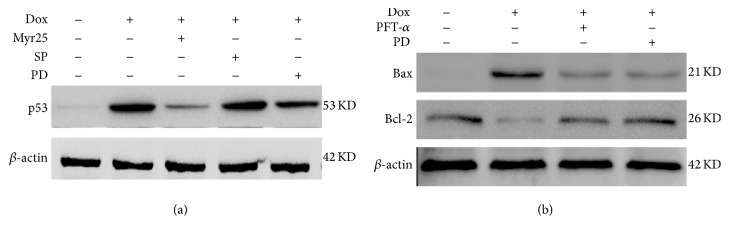
Involvement of ERK1/2 phosphorylation in myricitrin-mediated expression of p53, Bcl-2, and Bax. H9c2 cardiomyocytes were pretreated with or without myricitrin (25 *μ*g/mL) for 12 h prior to Dox exposure. Effects of SP600125 (a JNK-specific inhibitor) and PD98059 (an ERK-specific inhibitor) on myricitrin-mediated activation of p53 were measured by immunoblotting assay (a). Effects of PD98059 (an ERK-specific inhibitor) and PFT-*α* (an p53-specific inhibitor) on the expression of Bcl-2 and Bax were measured by immunoblotting assay (b).

Previous studies showed that ERK1/2 and JNK are associated with the activation of p53, which is assumed to regulate the expression of Bcl-2 and Bax, and the subsequent mitochondrial apoptosis in myocardial cells [11, 31]. Therefore, we first determined whether ERK1/2 or JNK was involved in the activation of p53 induced by Dox. The H9c2 rat cardiomyocytes were pretreated with an ERK1/2 inhibitor (PD98059) or a JNK inhibitor (SP600125) for 1 h before the addition of Dox, and the expression of p53 was measured. The results demonstrated that inhibition of ERK1/2 decreased the p53 expression. However, inhibition of JNK did not influence the Dox-induced p53 expression (Figure 9(a)). These results implied that Dox activated p53 in the H9c2 cardiomyocytes through the ERK1/2 pathway rather than JNK pathway. Next, we further demonstrated that ERK/p53 is upstream of Bcl-2 and Bax using the ERK1/2 inhibitor (PD98059) and a p53 inhibitor (PFT-*α*). As shown in Figure 9(b), inhibition of ERK1/2 and p53 prevented the Dox-induced upregulation of Bax and the downregulation of Bcl-2.

### 3.9. Myricitrin Had No Influence on the Antitumor Effect of Dox

**Figure 10:**
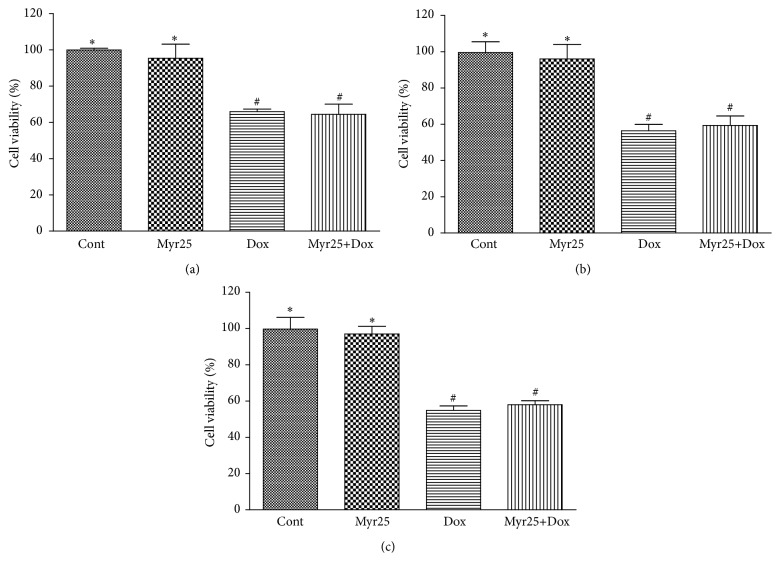
Effects of myricitrin on antitumor ability of Dox in vitro. Various cancer cell lines including HepG2 (a), Hep2 (b), and MCF-7 (c) cells were treated with Dox (1 *μ*M) in presence or absence of 25 *μ*g/mL myricitrin for 12 h prior to exposure to Dox for 36 h. The cell viability was measured by CCK8 assay. All data are expressed as the means ± SD (*n* = 3). ^#^ *P* < 0.05 versus Cont; ^*∗*^ *P* < 0.05 versus Dox-treated cells.

To determine whether myricitrin affected the antitumor action of Dox, several cancer cell lines including HepG2, Hep2, and MCF-7 cells were used for this experiment. As shown in Figure 10, Dox significantly decreased the viability of the cancer cells, but pretreatment with myricitrin did not influence this antitumor effect of Dox. Furthermore, myricitrin alone within the experimental concentration range did not significantly affect the growth of these cancer cells.

## 4. Discussion

Dox is a quinine-containing anthracycline antibiotic that is used to treat various forms of cancer, including hematologic and solid malignancies. However, the clinical use of Dox is limited by the common side effects of dose-dependent chronic cardiomyopathy or congestive heart failure [7]. Therefore, finding a potential compound from natural products that can decrease the cardiomyocyte cardiotoxicity without reducing the anticancer efficacy is a promising approach to improve the clinical application of Dox. Myricitrin is a natural flavone derived from* Myrica rubra* and other natural plants, which reportedly exhibits numerous biological activities such as antioxidative, anti-inflammatory, and antinociceptive effects. Our laboratory previously reported that myricitrin, which is isolated from the ground bark of* Myrica rubra*, prevented atherosclerosis by inhibiting oxidative stress-induced endothelial damage [21, 22]. However, its cardioprotective properties and the underlying mechanisms are still unknown. In this study, a series of experiments were designed to clarify whether the antioxidant potency of myricitrin contributed to the amelioration of cardiotoxicity induced by Dox.

The findings demonstrated that myricitrin possesses significant cardioprotective effects both in vivo and in vitro. We set up a chronic cardiac injury model in which rats were injected i.p. with Dox for a cumulative dose of 9 mg/kg. This dosage is just above the clinical threshold at which Dox-induced cardiotoxicity is expected to occur [32]. In this experimental model of Dox-induced injury to the myocardium in rats, pretreatment with myricitrin reduced the cardiac index and decreased the levels of serum cardiac enzymes including LDH, CK, and AST. Myricitrin also clearly alleviated the pathological changes and the histological damage in the heart tissues. In accordance with the in vivo results, pretreatment with myricitrin also effectively protected H9c2 cardiomyocytes from Dox-induced cell death. All of these results suggested that myricitrin exerts a prominent protective action against Dox-induced myocardial injury.

Although the exact mechanism of Dox-induced cardiotoxicity remains unknown, the ROS generated by Dox and the subsequent cardiomyocytes apoptosis are believed to be important factors [33–35]. The target organelles for Dox toxicity in cardiomyocytes are the mitochondria, based on the fact that mitochondrial enzymes activate Dox to the corresponding semiquinone, which undergoes redox cycling in air to generate ROS such as H_2_O_2_ and superoxide anion (O_2_^−•^) [14, 36]. The excess generation of ROS is thought to cause morphological and functional damage to the mitochondria and ultimately to induce DNA degradation and cardiomyocyte apoptosis through various pathways [37–39]. The present study demonstrated myricitrin inhibited the Dox-induced oxidative stress by reducing the creation of cellular ROS and activating the activity of SOD, CAT, and GSH-Px, three well-known antioxidases. In addition, the Dox-induced toxicity mediated through DNA damage in the myocardial cells was confirmed using the TUNEL assay. Through microscopic examination, typical nuclear and cellular morphological features of apoptosis were found in both Dox-treated heart tissues and H9c2 cardiomyocytes. However, myricitrin effectively reduced the number of TUNEL-positive cardiomyocytes. These results suggested that protective action of myricitrin against Dox cardiotoxicity is associated with its inhibitory effects on oxidative stress and myocardial apoptosis.

Apoptosis can be initiated by either the intrinsic (mitochondrial) or the extrinsic (death receptor-dependent) pathways [25]. Evidence has demonstrated that Dox directly activates the mitochondria-dependent apoptotic pathway and initiates the collapse of mitochondrial transmembrane potential (Δ*ψ*_*m*_). The disruption of Δ*ψ*_*m*_ leads to the release of proapoptotic molecules such as cytochrome c from the intermembrane space to the cytoplasm. There, cytochrome c forms an apoptosome with Apaf-1 and caspase-9, which promotes the activation of caspase-3 [40–42]. Caspases are a family of aspartate-specific cysteine proteases that play key roles in regulating apoptosis: of these, caspase-3 has been identified as the primary executioner of apoptosis [29, 43]. In the present study, myricitrin pretreatment stabilized the Dox-induced mitochondrial dysfunction, reduced the release of cytochrome c from the mitochondria, and inhibited the increased expression of cleaved caspase-9 and caspase-3. Together, these results showed that the antiapoptotic effect of myricitrin was associated with the mitochondria-dependent apoptotic pathway.

The tumor suppressor p53 is a potent transcription factor that plays a central role in the mitochondria-dependent apoptotic pathway [44]. Many reports have suggested that the mechanism of cardiac apoptosis induced by Dox is dependent on the activation of tumor suppressor p53. In one study, pifithrin-*α* (PFT-*α*), a chemical inhibitor of p53, was shown to protect against Dox-induced apoptosis and acute cardiotoxicity in mice [38]. In another study, transfection of p53 into ventricular myocytes was shown to initiate a mitochondria-dependent apoptotic pathway [45]. Various genotoxic stresses including those provoked by DNA-damaging agents result in the rapid activation of p53. Although the mechanism of the p53-dependent apoptosis is still not fully understood, it appears that this effect involves the transcriptional activation of many target genes including members of the MAPK and Bcl-2 families [46, 47]. MAPK consists of three major signaling cascades: extracellular signal-related kinases (ERK1/2), c-Jun N-terminal kinases (JNK), and p38 kinase (p38). It has been reported that the phosphorylation of ERK1/2 and JNK contributes to the p53-dependent cardiomyocyte apoptosis and the heart failure that is ultimately induced by Dox [11, 48, 49]. The present study indicated that myricitrin pretreatment neutralizes the Dox-induced phosphorylation of ERK1/2 and JNK. In a pharmacological approach, both an ERK-specific inhibitor (PD98059) and a JNK-specific inhibitor (SP600125) significantly inhibited the Dox-induced production of cleaved caspase-3 and the decrease in the cell viability. However, only the ERK-specific inhibitor (PD98059) suppressed the p53 expression. These findings suggested that myricitrin may modulate p53 signaling through ERK-dependent pathways.

Mice deficient in p53 exhibit increases in Bcl-2 and decreases in Bax protein levels, which suggests that the expression of Bcl-2 family genes may be directly regulated by p53 [50]. The Bcl-2 family contains both proapoptotic and antiapoptotic proteins that are recognized as key regulatory components of the mitochondrial apoptosis pathway [51]. Bcl-2 and Bcl-xl are the principal Bcl-2 family proteins that protect cells from apoptosis. They combine with Bax, a proapoptotic protein, to prevent its oligomerization. The oligomeric form of Bax promotes the loss of mitochondrial membrane integrity and causes the release of cytochrome c [52]. Other proapoptotic proteins, such as Bad, compete for binding to Bcl-2 or Bcl-xL. This causes release of Bax, which induces apoptosis. Thus, the balance between pro- and antiapoptotic Bcl-2 family proteins influences the rate of apoptosis [53, 54]. Our findings demonstrated that Dox treatment induces increases in Bax and Bad expression and decreases in Bcl-2 and Bcl-xl expression. Myricitrin blocked these changes and enhanced the Bcl-2-to-Bax ratio in H9c2 cardiomyocytes exposed to Dox. In a subsequent study, we used pharmacological inhibitors to investigate whether ERK/p53 is upstream of Bcl-2 and Bax in the Dox-induced cardiomyocyte apoptosis. The results showed that the ERK1/2 inhibitor (PD98059) and the p53 inhibitor (PFT-*α*) prevented the upregulation of Bax and downregulation of Bcl-2 induced by Dox. All of these results indicated that the cardioprotective effect of myricitrin was associated with the inhibition of ERK/p53 pathway and subsequent Bcl-2 family-mediated mitochondrial apoptosis.

Another issue of concern in this study was the effect of myricitrin on the antitumor activity of Dox. Our in vitro studies demonstrated that myricitrin pretreatment did not have a negative influence on antiproliferative effects of Dox in a series of cancer cell lines (i.e., MCF-7, HepG2, and Hep2 cells). These results may be explained by the fact that the cytotoxic mechanism of Dox is attributed to its inhibition of the DNA topoisomerases of neoplastic cells [55], which is entirely different from the mechanisms of side effects of Dox on myocardial cells.

## 5. Conclusions

In summary, the present study revealed a protective role for myricitrin in Dox-induced cardiotoxicity in vivo and in vitro. The underlying mechanism was associated with the antioxidant activity of this compound and its inhibition of the mitochondria-dependent apoptotic signaling mediated by ERK/p53 pathway. Our study provided direct evidence that myricitrin can be a novel candidate for clinical treatment to protect against Dox-induced cardiotoxicity.
